# Optimization of 3D printer settings for recycled PET filament using analysis of variance (ANOVA)

**DOI:** 10.1016/j.heliyon.2024.e26777

**Published:** 2024-02-27

**Authors:** Ciara O'Driscoll, Olamide Owodunni, Umar Asghar

**Affiliations:** School of Engineering, University of Wollongong Dubai, UOWD Building, Dubai Knowledge Park, Dubai, P.O.Box 20183, United Arab Emirates

**Keywords:** Additive manufacturing (AM), 3D printing, Cross sectional area (CSA) profiles, rPET, Sustainable filament, Mechanical properties, ANOVA, Ultimate tensile strength (UTS), Printing parameters

## Abstract

Fused Deposition Modeling (FDM) 3D printing creates components by layering extruded material. Printer parameters such as layer height and infill density can greatly impact the mechanical properties and quality of the printed parts. One critical factor to be considered in analysis is the anisotropy nature of printed components, considering all cross-sectional area (CSA) profiles for less than 100% infill density. This paper investigates the effect of the anisotropy nature of 3D printed CSA has on stress calculations and hence mechanical properties of the specimen through Design of Experiment (DOE). Analysis of variance (ANOVA) is utilised to evaluate the results. Printed specimens were tensile tested as per ASTM D638-14. Raw data was analysed using various CSA profiles, taking changes in infill density and layer height into account. Fixed parameter such as shell count, top and bottom layers, nozzle diameter, Hexagonal pattern were defined. Specimens Ultimate Tensile Strength (UTS) values increased on average by 30% using average profile CSA data compared to using external specimen dimensions. Further analysis assessing printer parameters affect on recycled Polyethylene Terephthalate (rPET) specimen's Young's Modulus (YM) and UTS was studied. One significant finding from this study suggests that the thickness of each layer has the most significant impact on the material properties of 3D printed rPET, as observed through the analysis of tensile test data obtained from 3D printed samples. A 3D printed rPET specimen with 30% infill density and 0.25 mm layer height has a higher YM (1175 MPa) and UTS (39 MPa) compared to a specimen with 75% infill density and 0.1 mm layer height (1159 MPa, 31 MPa). However careful interpretation of the results is required because for the same 30% infill parameter at 0.2 mm layer height the YM (936 MPa) and UTM (28 MPa) are significantly lower than at 0.25 mm layer height. If a higher value of YM and UTS is required an infill setting of 50% and layer height of 0.25 mm gave the highest values, YM (1330 MPa) and UTS (43 MPa).

## 1 Introduction

PET also known as Polyethylene Terephthalate, is a clear plastic that is mainly used for bottled packaging of soft drinks, juices and water. This makes it a single use item with potentially no alternative use. However, in recent years advancements have been made in using recycling plastic bottles for a variety of applications. One potential usage for recycling PET is for use in additive manufacturing, which is also referred to as 3D printing. Using recycled PET (rPET) for 3D printing is a relatively new material choice and a recent trend in research is to investigate suitability of recycled materials for 3D printing. This would allow single use PET to be removed directly from waste streams. Plastic waste represents a significant amount of municipal solid waste, with food and beverage packing and containers attributing to about 80% of thermoplastic waste. Numerous research studies have been conducted on recycling the waste stream from 3D printing, focusing on support structures, filament ends, and scraps from commonly used materials like Polylactic acid (PLA) and Acrylonitrile butadiene styrene (ABS). However, there is limited research on the slicer settings of recycled filament material [1,2]. This research aims to investigate if non-modified rPET, such as consumer plastic bottles, can be used for 3D applications by optimizing the printer slicer settings.

Slicer settings dictate how the design will be printed and they effect the material properties of the printed part. The main slicer settings are layer height, print speed, infill density and nozzle temperature. In a recent online article, the author stated that slicer settings are important, but also noted that different printers and materials will always require different settings to achieve a good print quality [3]. Several research studies on mechanical properties of 3D printed PLA samples have used print speeds ranging between 20 mm/s and 80 mm/s [[4], [5], [6], [7], [8], [9], [10], [11]]. The researchers mentioned either found no statistical significance of varying print speeds on UTS or they used fixed values for the research [[4], [5], [6], [7],^10^]. While similar research showed that higher print speeds caused voids and gaps in the printed material reducing the UTS of the samples [8]. Research analysis of 3D printed PET/rPET/PETG (Polyethylene terephthalate glycol is a modified version of PET creating a less brittle material for 3D printing) samples, details print speed values between 5 mm/s and 50 mm/s, with optimum speed cited at 20–30 mm/s [4,12,13]. Printer bed temperature has been widely reported and fixed for various filament materials. Research suggests that a heated printing bed should be used when using HDPE filament to reduce the effects of warping [14]. Print bed temperatures have a wide range (70 °C–100 °C) depending on the filament material, [4,15].

A study was conducted on the effect of layer height on the energy absorption levels and impact behaviour of PETG [16]. The authors found that layer height had a more significant effect than test temperature on experimental results from impact testing [16]. The study showed that a lower layer height of 0.1 mm exhibited a maximum absorbed energy value of 67.3 kJ compared to a sample with layer height of 0.4 mm with an absorbed energy of 40 kJ, which can be an indication of a stronger material using 0.1 mm layer height [16]. Reflow, a company focused on producing recycled filaments, suggest that when using recycled PETG (rPETG) a heated print bed of 70 °C should be used [15]. Mechanical properties of PETG have also been investigated while varying infill densities from 20 to 100% [17,18]. The researchers showed a gradual increase in UTS for increasing %infill density using a fixed layer height of 0.1 mm and Grid infill pattern [17,18]. Research with PETG samples showed that by increasing infill density from 20% to 80% can increase the value of UTS by up to 14% [18].

A study on recycled materials optimization showed that for a 4 mm rPET pellet to achieve the best adhesion results the ideal printer nozzle temperature setting should be 220 °C and 230 °C and bed temperature of 100 °C [4]. A conclusion from the study showed that material strength is not significantly affected by recycled material compared to virgin material [4]. Tensile Strength (UTS) of PET samples ranged from 27 to 45 MPa while rPET was determined to have an average UTS of 40 MPa [4]. Previous work reports average UTS of 3D printed rPET samples to be 29.74 MPa and average Young's Modulus (YM) of 3346 MPa [19]. The authors only detailed nozzle temperature 210 °C and nozzle diameter of 0.4 mm for 3D printer settings [19]. Their findings for UTS align with literature. However, the data for YM is significantly higher than other reported figures [[19], [20], [21]]. A study conducted on rPET as a sustainable material filament source for 3D printing and reported values similar to Ref. [4], 3D printed rPET UTS to be 24 MPa and YM of 1630 MPa [20]. They used a bed temperature of 70 °C, nozzle temperature of 255 °C, layer height of 0.15 mm and infill density of 100% printer settings, while noting the material used is 65% rPET with 35% virgin PET [20]. Compression testing was carried out by the authors, as per ATSM D695-15, for several patterns under the previous mentioned printer setting conditions. The testing included sample specimens printed in the x-y plane and the z-direction. The authors concluded the 100% infill with perpendicular pattern direction between printed layers, yielded highest YM results relative to the load criteria for both directions (z and x-y) of printing, while noting any variation in printing parameter can affect the mechanical properties of the material being printed [20]. Another slicer parameter widely mentioned is the printer infill density setting. A study investigating the effect of infill parameter during compression testing indicates that the compressive strength of the 3D-printed PLA samples increased with increasing infill density percentage [22]. The authors noted that with an increase in infill density reflects in an increase in the sample printing time [22].

Researchers have shown raster orientation and shell number not to be statistically significant in relation to UTS of 3D printed PLA specimens [5,23]. However, a study states that samples with 100% infill density are subject to influence from raster orientation [6]. Research concluded that build orientation of 45° is optimum for ultimate UTS of FDM printed PLA tensile samples. However the results indicate only a very small difference in comparison to build orientation of 0° (printed flat on x-y plane), 38.92 MPa versus 39.44 MPa for 45° (printed at an angle of 45° to x-y plane) [23]. Results from recent literature showed the build orientation of the tensile sample being printed ‘on-edge’ on the x-y plane was optimum for higher values of UTS of PLA samples [5]. However, other research findings state that although printing on-edge provides higher UTS results, flat printing samples provides a better possibility of predicting the effects of other print parameters [6]. A recent study has concluded that smaller layer heights contributed to less voids in the printed material leading to the observation that UTS decreases with increasing layer thickness [23]. Research has shown for higher values of infill 60% and 90% of PLA printed samples that layer height has little effect on UTS [24]. Several researchers have shown that 100% infill gives the highest values for UTS and better mechanical properties of PLA printed samples [5,6,23]. Other researchers fix infill parameter at 100% for both PLA and ABS samples [8,[25], [26], [27]]. Research showed that for PLA samples 100% infill showed brittle-like behaviour compared to 15% hexagonal pattern infill [28]. The research also showed a higher value of UTS for the lower infill pattern 15% than that of 100% solid sample [28]. Researchers have shown that increasing % infill density has resulted in an increase in UTS of sample data [5,17,29,30]. While some researchers have analysed the effects of porosity and voids within the printed material profiles [7,30]. Few studies have discussed the anisotropy of the printed cross-sectional areas (CSA) of specimens with infill patterns below 100% infill density. Several studies detail CSA with images or mention lower infill will result in reduced CSA however there is no mention if the variation of CSA between samples was considered when calculating the mechanical properties [6,7,17,23,27,29].

Further research on the variation in CSA and its impact on mechanical properties is required and is detailed in this research paper. This study aims to analyse the effect of various 3D printer settings on rPET filament material properties through application of Design of Experiment (DOE) and analysis of variance (ANOVA).

## 2 Materials and methods

### 2.1 Material

**Fig. 1:**
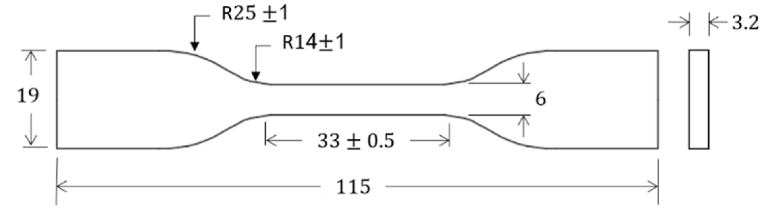
Tensile test sample as per ASTM D638-14, (units mm).

**Fig. 2:**
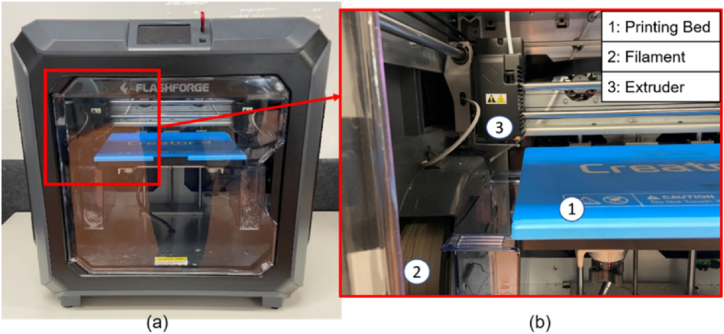
(A) Flashforge opensource FDM 3D printer (b) close-up of printer area showing 1: printer bed; 2: filament and 3: extruder.

In this research, rPET filament material (Table 1) was used to 3D print tensile test samples as per ASTM D638-14 (Fig. 1) using Flashforge opensource FDM 3D printer, shown in Fig. 2 (a). Fig. 2 (b) illustrates the 3D printer bed, spooled filament material and extruder component. Critical 3D printer settings identified during the literature review namely; nozzle temperature, layer height, infill density, print speed, infill patterns are further outlined in Section 2.5. Two batches of rPET filament material were used during this research namely rPET_1 and rPET_Ulrafuse [21] shown in Table 2. Initial mechanical testing; T1 (Table 3) was undertaken with rPET filament material created by UOWD, using single source recycled PET water bottles; rPET_1 (Table 2).^1^ Further mechanical testing; T2 (Table 3) was carried out on rPET filament that was purchased from BASF 3D Printing Solutions BV (created from recycled PET water bottles); rPET Ultrafuse® (refer to Table 2).

**Table 1:** Tensile test specimen 3D printer settings for rPET filament materials.

		Filament material Name		
	Slicer settings		rPET_1^1^	rPET Ultrafuse® 2
**A**	Nozzle Temperature	(°C)	240	235
**B**	Layer height	(mm)	0.2	Not provided
**C**	Infill density	(%)	50	Not provided
**D**	Print Speed	(mm/s)	30	40

**Table 2:** Material properties of rPET filament materials. Both materials are created using only recycled PET water bottles as a source material with filament extrusion diameter of 1.75 mm.

Filament Name	Created/purchased	Tensile Strength (MPa)	Young's Modulus (MPa)	% Elongation at break
rPET_1	Created	31.3 MPa	744 MPa	6.5%
rPET Ultrafuse®	Purchased	38.6 MPa	1640 MPa	4.2%

**Table 3:** Tensile testing methodology.

Test	Material	Experimental details	Analysis method	Number of runs	Specimen per run	Test repeated (per sample)	Output (MPa)
T1	rPET_1	Table 4: **Slicer settings for T1**	2^4^ factorial design	16 (refer to Table 4)	2	×3	Young's Modulus (E)
						Once, test to failure	Tensile Strength (UTS)
T2	rPET Ultrafuse®	Table 7: **Slicer settings for T2**	4^2^ factorial design	16 (refer to Table 7)	2	×3	Young's Modulus (E)
						Once, test to failure	Tensile Strength (UTS)

### 2.2 CAD model preparation

**Fig. 3:**
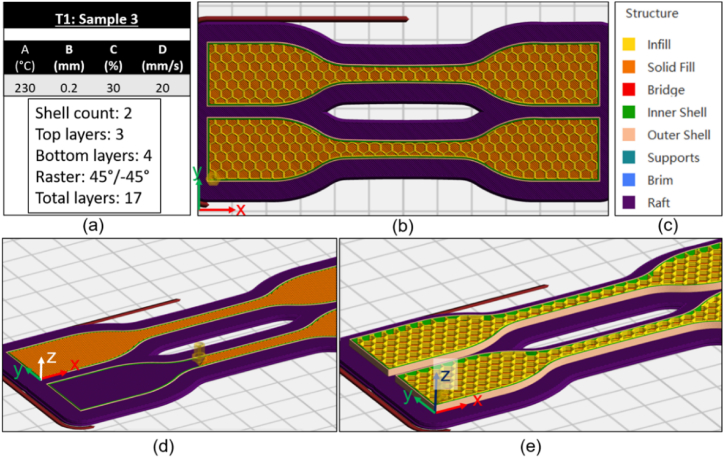
T1 Sample 3 (a) Slicer parameters (b) Top view of Hexagonal infill pattern (c) colour key (d) view of outer wall shell count of two (e) view of inner infill pattern Hexagonal pattern with 30% infill density. (Colour is required). (For interpretation of the references to colour in this figure legend, the reader is referred to the Web version of this article.)

**Fig. 4:**
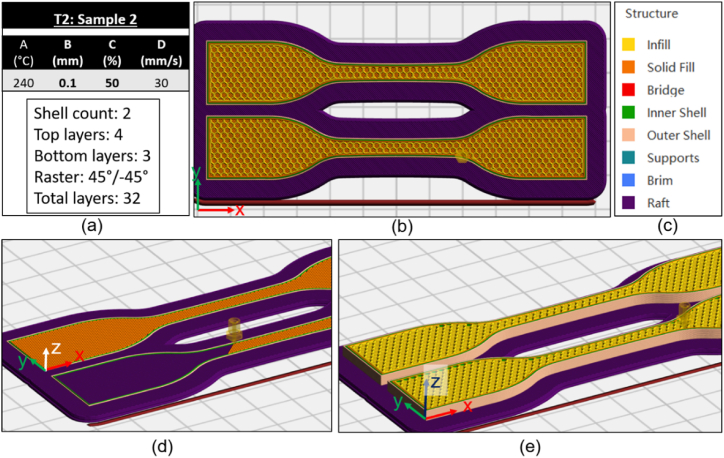
T2 Sample 2 (a) Slicer parameters (b) Top view of infill pattern (c) colour key (d) view of outer wall shell count (e) view of inner infill pattern. (Colour is required). (For interpretation of the references to colour in this figure legend, the reader is referred to the Web version of this article.)

Tensile test samples were designed using Autodesk Fusion 360 CAD software and converted to G-code files using Flashprint slicer software, refer to Fig. 3 (a)–(e) and Fig. 4 (a)-(e) for specimen sample printing parameters and G-code images. Build orientation, raster orientation, number of shells (specimen wall), top and bottom layers can all be set within the Flashprint slicer software, Figs. 3 (a) and Fig. 4 (a). Settings were selected based on current literature outlined in Section 1. Solid (100% infill) top and bottom layers were added to the slicer settings using 45°/-45° raster angle (Fig. 3 (a), (d) and Fig. 4 (a), (d)), this aligns with current research [31]. To investigate infill density as a parameter for rPET samples, neither extreme 0% or 100% was selected. The infill density minimum and maximum percentages was set at 30% and 50% respectively for initial testing (T1 refer to Table 4). As previously stated many studies of PLA, ABS and PETG printed samples have fixed infill at 100%, while other studies use range values from minimum of 20% infill density [13,17,18,29]. Researchers show 50% infill as optimal setting for PLA and PETG materials under tensile loading [13]. Maximum value of 50% was selected to reduce the material used, reduce the time to print and analyse the interactions with other selected parameters. The minimum value of 30% was selected to reflect a lower infill % for rPET material. Layer height was set to minimum machine setting of 0.1 mm and the current research used the slicer software default setting of 0.2 mm (50% of filament nozzle diameter) for the maximum setting. Specimens were printed using Hexagonal pattern, Figs. 3(b)–Fig. 4 (b).

**Table 4:** Slicer settings for T1; 2^4^ factorial design.

			Level 1	Level 2
Factors	Slicer settings		Low setting (−)	High setting (+)
**A**	Nozzle Temperature		230	240
**B**	Layer height		0.1	0.2
**C**	Infill density		30	50
**D**	Print Speed		20	30

### 2.3 Specimen fabrication

**Fig. 5:**
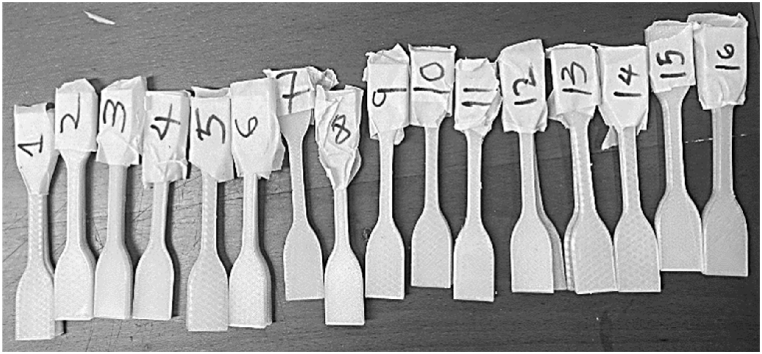
32 T1 tensile samples after printing.

Tensile test samples were printed in the flat (x-y plane) as per slicer setting detailed in Table 4. Specimen dimensions are based on ASTM D638-14 Type IV 115 mm length, with the narrow section 6mm wide by 3.2 mm thick by 33 mm length gauge length, shown in Fig. 1. Fig. 5 shows T1 tensile samples after printing.

**Fig. 6:**
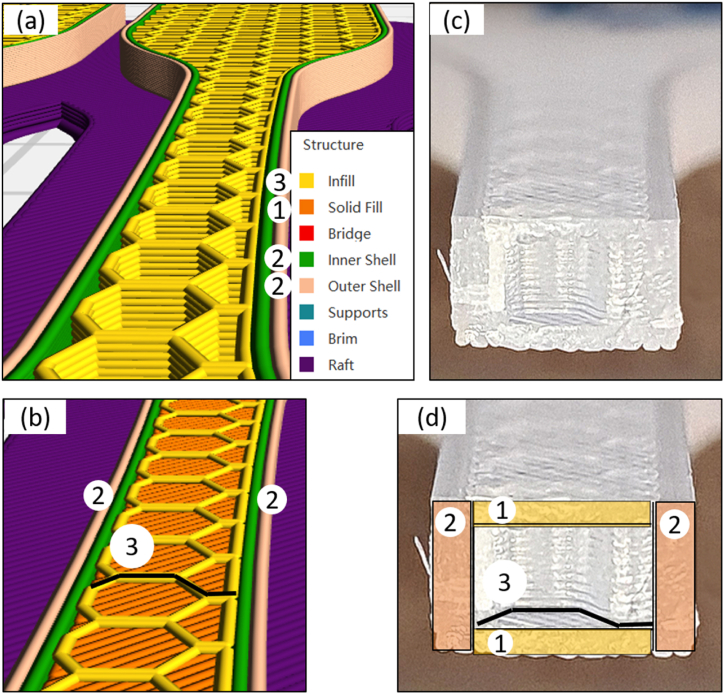
Cross sectional area of a tensile test sample (a) Slicer images showing shell (inner and outer) and infill pattern (Hex 30%) (b) Slicer image showing bottom layer (45°/-45° raster angle), shell (inner and outer) and infill pattern (Hex 30%) (c) Actual tensile sample after fracture (d) Actual tensile sample after fracture detailing the print slicer parameter 1–3. (Colour is required). (For interpretation of the references to colour in this figure legend, the reader is referred to the Web version of this article.)

It should be noted variation of cross-sectional area (CSA) occurred in actual printed samples. All specimens were measured using a digital vernier calliper prior to tensile testing. Printer pattern selected was Hexagonal for T1 and T2. The printed pattern can be seen in Fig. 6 (a).

A sample cross sectional area (CSA) can be seen in Fig. 6 for T1 sample after fracture. Note this image is post fracture hence deformation has occurred to the sample shown in Fig. 6 (c) and (d) however the profile of the Hexagonal pattern (reference 3: black line in Fig. 6) can be clearly seen for comparison to image from the slicer software Fig. 6 (b). The sample specimen is Hexagonal pattern with 30% infill density and has fractured as per the figure. The profile of the CSA can be divided into fixed parameter profile settings Fig. 6 (d) reference 1: top and bottom layers with 45°/-45° raster angle, are visible post fracture and reference 2: the two-wall shell count. CSA data was calculated for each specimen based on layer height and infill density parameters used to calculated stress values. Taking porosity or voids in the material into account is outside the scope of this research.

### 2.4 Cross sectional area (CSA) profiles

**Fig. 7:**
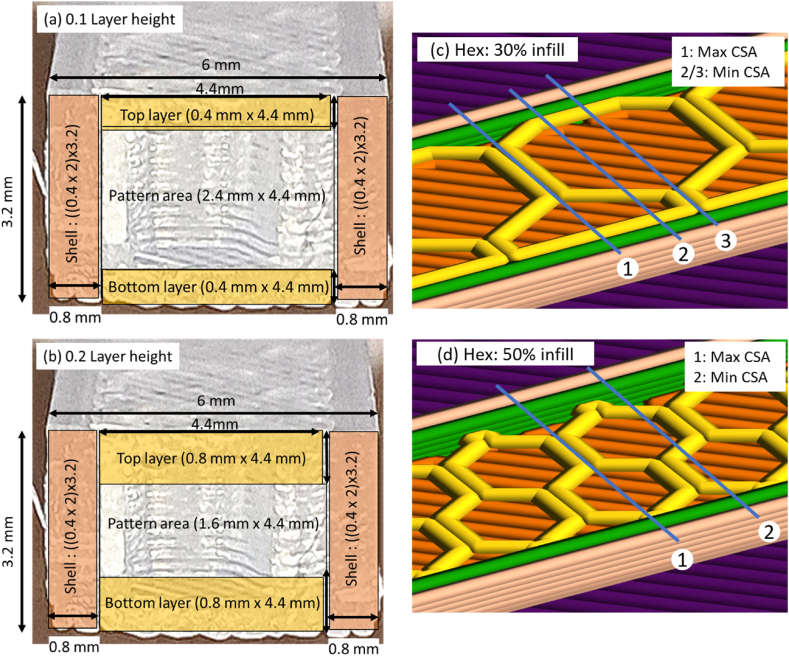
Sample CSA calculations based on fixed and variable parameters (a) Fixed parameters and variable pattern area for 0.1 Layer Height (b) Fixed parameters and variable pattern area for 0.2 Layer Height (c) Hexagonal pattern with 30% infill density showing approximate CSA profiles for a maximum and minimum area (d) Hexagonal pattern with 50% infill density showing approximate CSA profiles for a maximum and minimum area. (Colour is required). (For interpretation of the references to colour in this figure legend, the reader is referred to the Web version of this article.)

Fig. 7 details the two profiles for this analysis. Fig. 7 (a) shows a Hexagonal pattern, 0.1 mm layer height with three bottom layers (Initial layer is 0.2 mm) giving a 0.4 mm height and four top layers, 0.1 mm x 4. Fig. 7 (a) shows a two-shell count wall where the nozzle diameter is 0.4 mm giving a 0.8 mm thickness. CSA for each highlighted section can be calculated based on CAD model dimensions 3.2 mm × 6 mm as per Fig. 1. Fixed CSA for 0.1 mm layer height is calculated as 8.64 mm^2^ while fixed CSA for 0.2 mm layer height is 12.16 mm^2^. Variation in the CSA profile occurs within the pattern area shown in is Fig. 7 (a) and (b). For 0.1 mm profile the remaining CSA is 2.4 mm × 4.4 mm. The same analysis has been applied to 0.2 mm layer height samples reducing the pattern CSA dimensions to 1.6 mm × 4.4 mm, Fig. 7 (b). Since the samples have been printed in the x-y plane (flat as detailed in Section 2.2) the CSA varies, Fig. 7 (c) & (d). Based on the infill density selected, for this research 30% and 50%, the profiles with maximum and minimum material across the cross section have been identified. The profile paths are shown in Fig. 7 (c) & (d). Analysis of properties such as YM and UTS have been calculated using four different profile parameters; 1: measured external CSA; 2: Maximum profile CSA; 3: Minimum profile CSA; 4: Average CSA taken from Max and Min profiles. CSA calculation for each sample is presented in Section 2.6.1.

### 2.5 Experimental testing

**Fig. 8:**
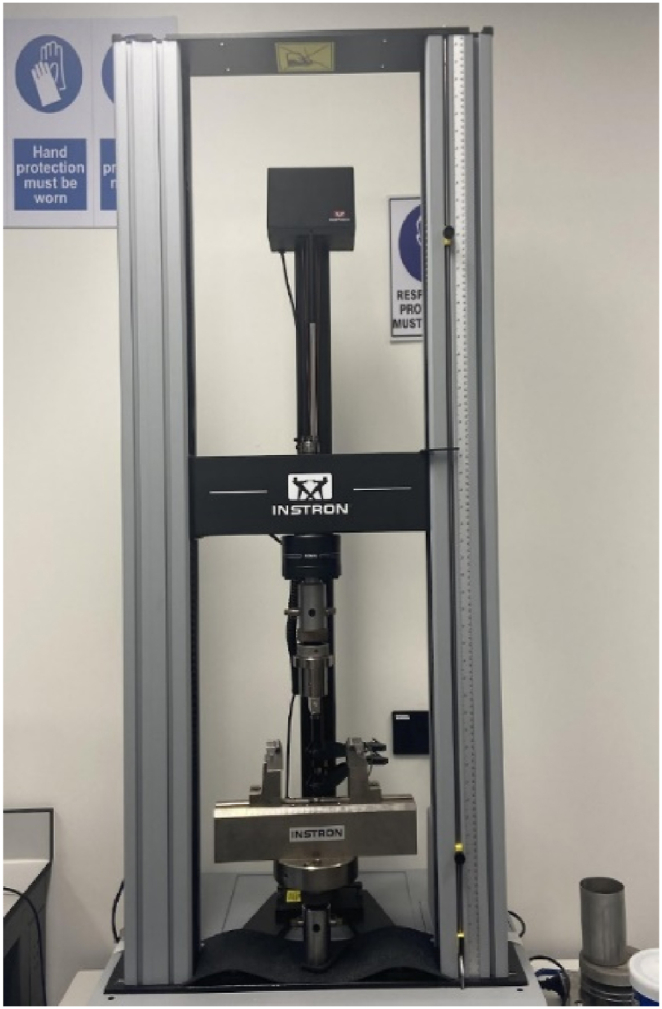
Instron Universal Testing Machine used to conduct all tensile testing.

All mechanical tests were conducted using Instron Universal Testing Machine (UTM) Fig. 8 as per ASTM D638-14. Mechanical load was applied using a 50 kN load cell and both load and deformation data were continually recorded by the UTM throughout the tensile testing.

In this research two sets of tensile tests were conducted namely; T1 and T2 refer to Table 3. The details of the mechanical tests such as material used, printer settings, number of samples and output responses are outlined in Table 3. Initial testing T1 was conducted as a baseline study on high and low selected parameters for rPET samples as detailed in Sections 1, 2.2. Based on the results obtained from T1 a second set of testing was developed to understand the interaction between two parameters namely; layer height and infill density.

Material properties including YM and UTS were determined for the 3D printed rPET samples. Experiments T1 and T2 had 16 different printer setting combinations. For each setting combination, two specimens (A and B) were printed and tested. Three tests were conducted per specimen within the elastic limit range, as determined by preliminary testing, to calculate YM. The tensile specimen was loaded to a maximum load of 300 N, the load was reversed to zero and this process was repeated twice without removing the specimen from experimental set-up. The specimens were removed from the experimental set-up and repositioned before tensile testing each specimen to failure to determine the UTS of the samples (full results are presented in Section 3.3).

### 2.6 Statistical analysis

This research will utilise statistical analysis to analyse rPET filament material property data from 3D printed samples YM and UTS under various test conditions outlined in Section 2.1 Table 3.

It was fundamental to consider the extent to which the sample means varied based on the CSA used to determine the properties YM and UTS. The statistical test is based on a statistic that possesses an F distribution and specifies that the hypothesis of no effect for the independent variable should be rejected if the value of F is large [32]. F statistic is a ratio. Its numerator and denominator are both estimates. Random samples have been drawn from k population means $\mu_{1},\mu_{2},\ldots \mu_{k}$, and common variance $\sigma^{2}$. The null hypothesis $H_{0}$, is that all the population means are equal:

$${H_{0}:\mu }_{1}=\mu_{2}=\ldots \mu_{k}$$

The alternative hypothesis is that not all means are equal.

$${H_{a}:\mu }_{i}\neq \mu_{j}\;for\;some\;i\;and\;j$$

The usual significance level is set at α=0.05. If the probability of error is greater than 0.05 (p > 0.05) then there is not enough evidence to reject the null hypothesis. The smaller the probability becomes, the more compelling the evidence that the null hypothesis should be rejected [32]. In this research, a 2^4^ factorial design ANOVA was used to identify the significant independent variable and determine how they affect the response data. ANOVA evaluates whether there are statistically significant differences in the means among two or more populations [33]. Statistical analysis was performed using Minitab® 21.4 statistical software.

#### 2.6.1 Experimental design, T1

A 2^4^ factorial design, DOE, was developed to study the relationship between various 3D printer settings and material property data. Table 4 shows the four identified slicer setting factors; A:nozzle temperature (°C), B:layer height (mm), C:infill density (%) and D:printer speed (mm/s). Two levels were used for this experiment, each factor has a low and high setting based on ranges identified during literature review. The initial nozzle temperature range was 220 °C–230 °C, however samples failed to print at the lower level 220 °C. The temperature was adjusted to 225 °C but again samples failed to print. The range was adjusted to lower level of 230 °C and an upper level of 240 °C. There was no issue with printing rPET sample at 240 °C. Two rPET tensile samples were tested for each run of the 2^4^ factorial DOE; each run has pre-defined slicer settings as outlined in Table 5. A total of 8 sets of data results were collected for T1, refer to Table 3.

**Table 5:** 2^4^ factorial design parameters.

		Sample Number															
Factors		1	2	3	4	5	6	7	8	9	10	11	12	13	14	15	16
**A**	**(°C)**	240	240	240	240	240	240	240	240	230	230	230	230	230	230	230	230
**B**	**(mm)**	0.1	0.1	0.1	0.1	0.2	0.2	0.2	0.2	0.1	0.1	0.1	0.1	0.2	0.2	0.2	0.2
**C**	**(%)**	30	30	50	50	30	30	50	50	30	30	50	50	30	30	50	50
**D**	**(mm/s)**	20	30	20	30	20	30	20	30	20	30	20	30	20	30	20	30

The maximum and minimum CSA profiles were calculated based on the profiles shown in Fig. 7. There are four factor (B:layer height * C:infill density) combinations that influence CSA profile calculations, namely; 0.1 mm & 30%; 0.1 mm & 50%; 0.2 mm & 30% and 0.2 mm & 50%, Table 6. For example; for 0.1 mm layer height and 30% infill density the max profile of the length of material was measured from CAD model slicer file as 2.6 mm. Hence the filled CSA for that profile was ${\mathrm{Max}}_{CSA}=2.4\times 2.6=6.24\;{mm}^{2}$. This is then added to 8.64 mm^2^ fixed CSA to get 14.88 mm^2^ shown in Table 6. The task was completed for all potential CSA profile paths to take the anisotropy of the printed samples into account.

**Table 6:** Printed samples and CSA profile parameters.

			Calculated CSA (mm^2^)	
Sample numbers	Factor B (mm)	Factor C (%)	Max	Min
1, 2, 9, 10	0.1	30	14.88	12.48
3, 4, 11, 12	0.1	50	13.20	12.24
5, 6, 13, 14	0.2	30	16.32	14.72
7, 8, 15, 16	0.2	50	15.20	14.56

**Table 7:** Slicer settings for T2; 4^2^ factorial design.

Factors	Slicer settings	Level 1	Level 2	Level 3	Level 4
**B**	Layer height	0.1	0.15	0.2	0.25
**C**	Infill density	30	50	60	75

#### 2.6.2 Experimental design, T2

Table 7 below details slicer settings for T2, focusing on two factors identified in T1 as significant; B:LH (mm) and C:ID (%). Level settings were selected to introduce smaller increments between levels. The reduction in UTS with increasing layer height was examined further by adding additional levels. All other printer settings were fixed for this set of testing; nozzle temperature 240 °C and print speed 30 mm/s; these two settings showed higher values of UTS in T1. The pattern was fixed at Hexagonal (same as T1) and the print bed was fixed at 100 °C. A total of 8 sets of data results were collected for T2, refer to Table 3.

## 3 Results and analysis

### 3.1 Preliminary tensile testing

**Fig. 9:**
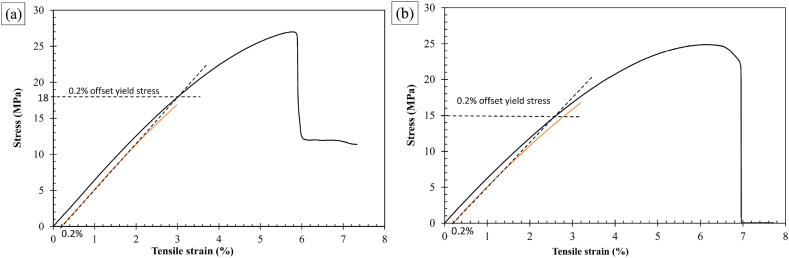
Example of tensile test data to establish yield point using 0.2% offset method (a) Nozzle Temperature 230 °C, Layer Height 0.1 mm, Infill Density 30%, Printer speed 20 mm/s and (b) Nozzle Temperature 240 °C, Layer Height 0.1 mm, Infill Density 30%, Printer speed 20 mm/s.

Preliminary testing was conducted using rPET_1 filament at the following printer settings: Bed temperature 100 °C, nozzle temperature 230 °C/240 °C, layer height 0.1 mm, infill density 30%, Printer speed 20 mm/s and Hexagonal Pattern to establish experimental set-up and material property values identifying the elastic and plastic ranges. The yield limit was determined to be between 15 MPa and 18 MPa stress, see Fig. 9 (a) and Fig. 9 (b). The lower limit of 15 MPa (300 N force) was set and initial tensile testing to determine the specimens YM values did not exceed this limit, Fig. 9 (b).

### 3.2 CSA profile data for T1 and T2 test samples

The CSA profile data is given in Appendix Table 1 for each profile detailed in Section 2.4 and Table 6, Section 2.6.1 for T1. The CSA profile data is provided in Appendix Table 2 for each profile detailed in Section 2.4 and Table 7, Section 2.6.2 for T2. The difference between the CAD profile CSA (CSA = 19.2 mm^2^, see Fig. 1) and measured CSA was added to the theoretical Max, Min and Avg calculations shown in Appendix Table 1, Table 2 to account for any difference in area of the printed samples. The data presented will be used in calculating mechanical properties from tensile tests.

### 3.3 Tensile testing experimental results

**Fig. 10:**
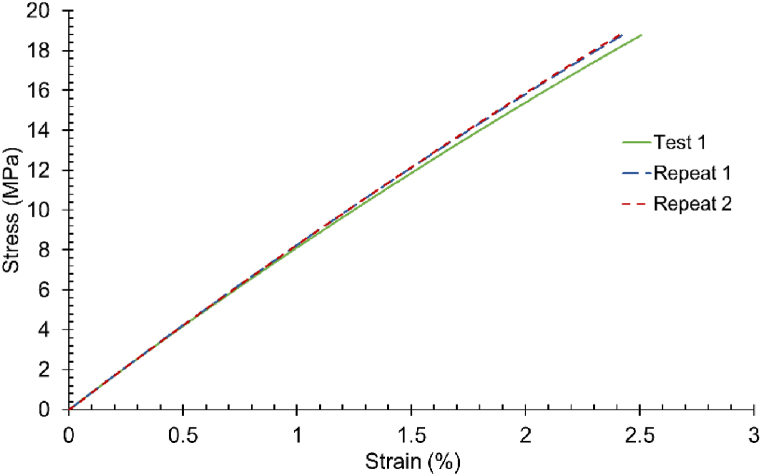
Stress-strain graphs for T1 sample. The tensile test was conducted three times without removing the sample from the experimental set-up.

An example of the output stress-strain graphs is shown in Fig. 10 using measured external CSA data.

Table 8 and Table 9 show an example (Sample 1 A) of the calculated YM values for each loading cycle and UTS found from testing samples to failure using the varying CSA profiles.

**Table 8:** Example of Young's Modulus (MPa) calculations with varying CSA profiles.

Experimental test: T1 Young's Modulus (MPa)				
CSA	Measured	Max	Min	Avg
**Test 1**	662.32	748.54	880.83	809.32
**Repeat 1**	669.05	776.40	913.61	839.43
**Repeat 2**	670.20	780.84	918.83	844.23

**Table 9:** Example of Tensile Strength (MPa) calculations with varying CSA profiles.

Experimental test: T1 Tensile Strength (MPa)				
	**Measured**	**Max**	**Min**	**Avg**
**Test to failure**	24.88	31.15	36.64	33.6

**Fig. 11:**
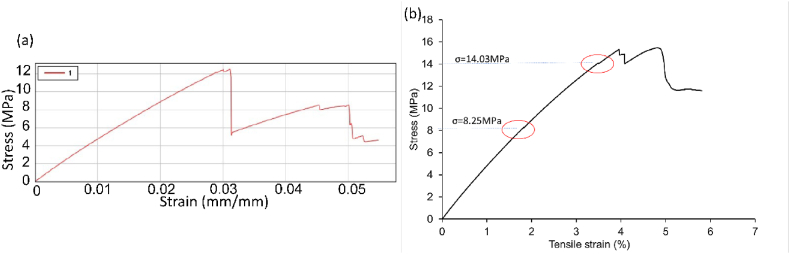
T1 (a) sample 11a (printer settings; Nozzle Temperature A:230 °C, Layer Height B:0.1 mm, Infill Density C:50%, Printer speed D:20 mm/s) stress-strain graph showing failure occurring in specimen before the predicted yield limit of 15 MPa. (b) Sample 11 b (printer settings; Nozzle Temperature A:230 °C, Layer Height B:0.1 mm, Infill Density C:50%, Printer speed D:20 mm/s) stress-strain graph showing two points at which the stress drops before increasing again.

During testing, sample number 11, factor settings A: 230 °C, B: 0.1 mm, C:50% and D:20 mm/s failed before expected stress resulting in lower UTS values, 12.58 MPa and 15.46 MPa respectively for measured CSA. These values increased to 22.12 MPa and 22.78 MPa using average CSA data. The tensile data graphs for T1 sample 11a and 11 b are shown in Fig. 11 (a) and Fig. 11 (b). Results from sample 11 are included in the factorial analysis.

**Fig. 12:**
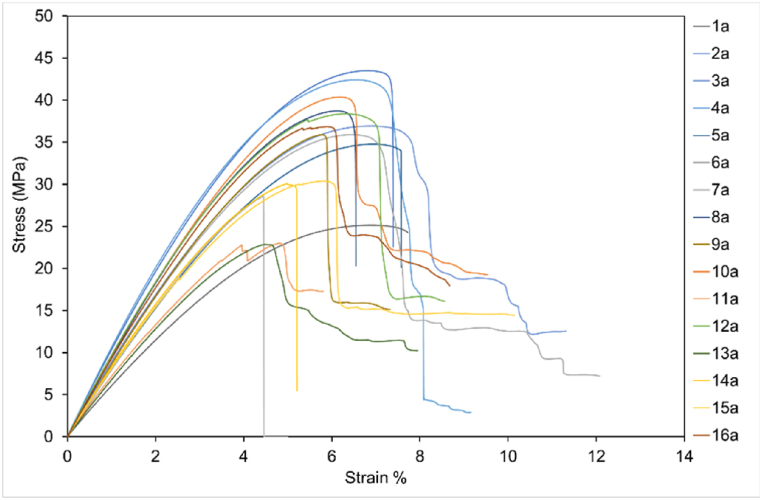
Stress-strain graph for T1 test data. (Colour is required). (For interpretation of the references to colour in this figure legend, the reader is referred to the Web version of this article.)

**Fig. 13:**
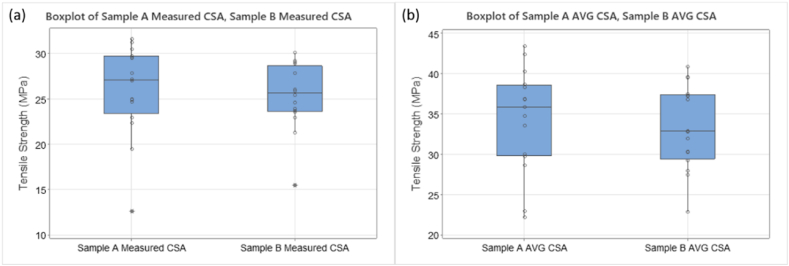
Boxplot data of Tensile Strength response for T1. (a) Sample 11 outlier data can be seen for both a and b data sets (b) Data using Avg CSA calculations, individual data points fall within whiskers.

**Fig. 14:**
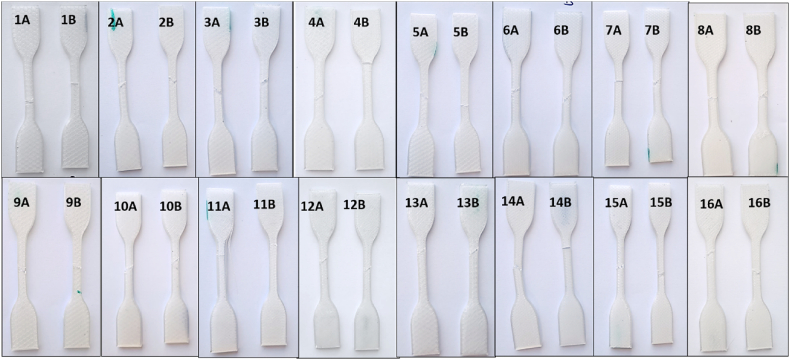
T1 samples after tensile testing.

Stress-strain data for one full set of results from T1 testing using average CSA data are shown in Fig. 12. The percentage strain ranges from 4.5% to 12% indicating ductile behaviour of rPET under certain printer settings. Fig. 12 also shows the range of UTS for rPET_1 3D printed samples, from 22 MPa to 43 MPa. The outliers for T1 data set, sample 11, can be seen in the boxplot data shown in Fig. 13 (a) with stress calculated using measured CSA not taking variation into consideration. Fig. 13 (b) shows values calculated using average CSA as detailed in Appendix Table 1. Fig. 14 shows T1 samples after failure.

**Fig. 15:**
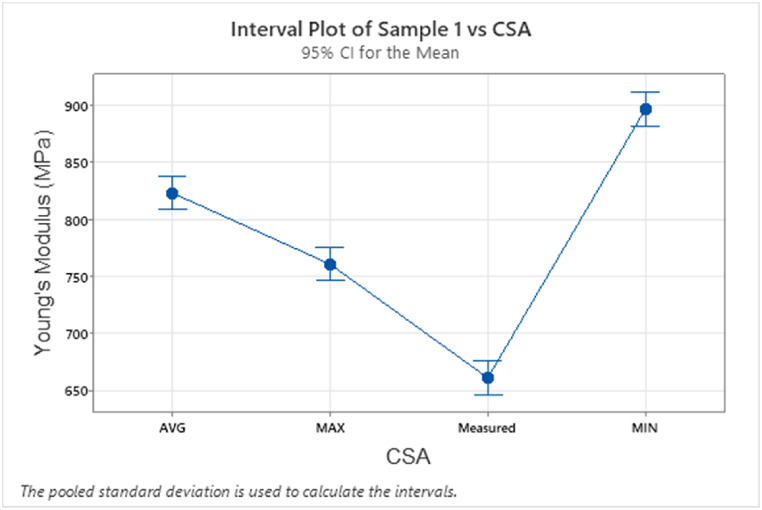
Interval plot of Young's Modulus data for sample 1 A with varying CSA data.

A statistical analysis was carried out on the YM values calculated using the variation in CSA, as detailed in Section 2.3, considering the anisotropy of 3D printed samples CSAs. The full YM data is provided in Appendix Table 3. ANOVA was used to determine whether the material property data was significantly different based on the CSA used. It is fundamental to consider the extent to which the means vary from one-another (between group variance) and the variability of the measurements within each group (within group variance). Excluding sample 11 which failed, all other sample data have been analysed. The one-way ANOVA showed that there is statistically significant evidence at α=0.05 to show that there is a difference in mean YM values taking the anisotropy of the printed sample into account, refer to Appendix Table 4. The critical F value is 3.0984 for ${df}_{1}=3\;and\;{df}_{2}=20$. The null hypothesis, $H_{0}$ should be rejected if the F statistic value is greater than 3.0984. A sample of the analyses data for sample 1 A is shown with F and *p* values, refer to Appendix Table 4. Fig. 15 shows the interval plot of the CSA data. From the analysis the null hypothesis can be rejected.

Because the measured CSA does not accurately reflect the internal cross section profile of less than a 100% infill density this data was removed from the analysis. The ANOVA was run again with the remaining three CSA data sets; Max, Min and Avg. The critical F value is 3.6823 for ${df}_{1}=2\;and\;{df}_{2}=15$. The null hypothesis, $H_{0}$ should be rejected if the F statistic value is greater than 3.6823. The analysis showed that for CSA profiles with 0.2 mm layer height and 50% infill density (Samples 7, 8, 15, 16, refer to Table 6 for profiles) there is not enough evidence to reject the null hypothesis, refer to Table 10 for ANOVA data. This result aligns with the profiles having a smaller remaining CSA due to 0.2 mm layer height and fixed parameters (Fig. 7). For all other sample data analysed, the null hypothesis was rejected (all *p* values < 0.05), meaning data means are statistically different.

**Table 10:** ANOVA CSA for Samples 7, 8, 15 and 16 showing null hypothesis cannot be rejected.

Sample 7: Analysis of Variance						Source	DF	Adj SS	Adj MS	F-Value	*P*-Value
Source	DF	Adj SS	Adj MS	F-Value	*P*-Value	CSA	2	3410	1705	1.38	0.282
CSA	2	2538	1268.8	1.58	0.238	Error	15	18 556	1237		
Error	15	12 035	802.3			Total	17	21 967			
Total	17	14 572				Sample 8: Analysis of Variance					
**Sample 15: Analysis of Variance**						**Sample 16: Analysis of Variance**					
Source	DF	Adj SS	Adj MS	F-Value	*P*-Value	Source	DF	Adj SS	Adj MS	F-Value	*P*-Value
CSA	2	2917	1458.3	1.75	0.208	CSA	2	3743	1871.5	3.27	0.066
Error	15	12 504	833.6			Error	15	8589	572.6		
Total	17	15 420				Total	17	12 332			

**Fig. 16:**
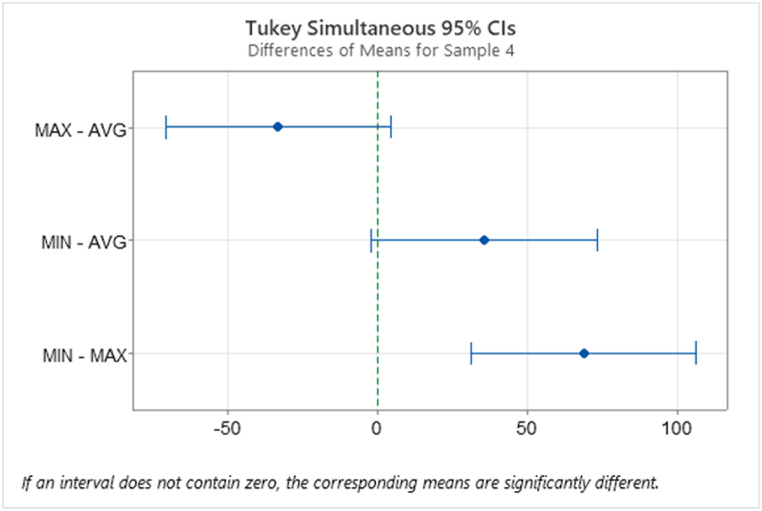
Tukey method with 95% Confidence difference of means plot for Sample 4 T1.

Using a Tukey Test on the remaining samples showed that Sample 4 had mean groups which were not significantly different, Table 11 and Fig. 16. For Sample 4 Tukey results there doesn't appear to be a correlation with the other samples with the same CSA profile (Table 6). Indicating the other printing parameters may be influencing the data. A full 2^4^ factorial design will be carried out using average CSA data.

**Table 11:** Tukey Test showing the mean groups that are not significantly different for Sample 4.

Sample 4: Grouping Information Using the Tukey Method and 95% Confidence			
CSA	N	Mean	Grouping
MIN	6	1030.5	A
AVG	6	995.1	A B
MAX	6	961.98	B

Sample 4: Tukey Simultaneous Tests for Differences of Means						
Difference of Levels	Difference of Means	SE of Difference	95% CI		T-Value	Adjusted *P*-value
MAX - AVG	−33.2	14.5	(-70.8, 4.4)		−2.29	0.088
MIN - AVG	35.3	14.5	(-2.2, 72.9)		2.44	0.067
MIN - MAX	68.5	14.5	(31.0, 106.1)		4.73	0.001

Means that do not share a letter are significantly different.

Individual confidence level = 97.97%.

### 3.4 T1 analysis

**Fig. 17:**
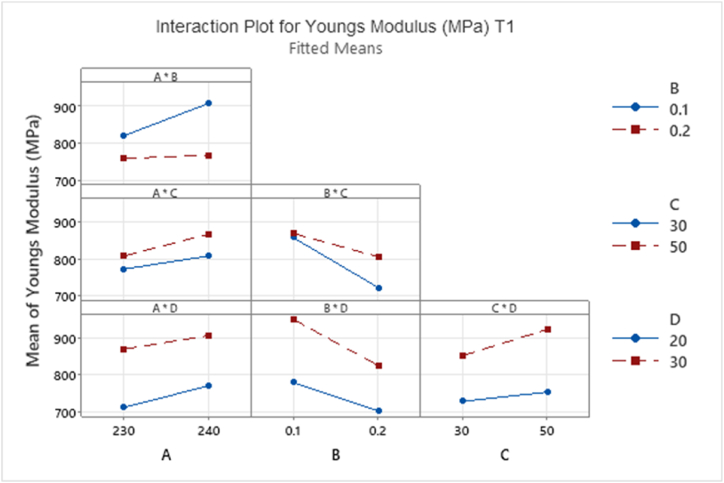
Interaction plot for factorial response data for Young's Modulus (MPa) T1. The four identified slicer setting factors; A:Nozzle Temperature (°C), B:Layer Height (mm), C:Infill Density (%) and D:Printer speed (mm/s).

ANOVA was run for the avg CSA data shown in Appendix Table 3. ANOVA results for T1 using YM values as the response data are shown in Appendix Table 5. Yellow highlighted main effects and interactions are significantly relevant with *p* values < 0.05. Significant main and interaction effects highlighted in yellow shows that all main effects A:NT (°C), B:LH (mm), C:ID (%) and D:PS (mm/s) and two-way interactions A * B and B * C are statistically significant, Appendix Table 5. Main effects should not be analysed in isolation hence the interpretation of these results must include the analysis of the interactions plots, Fig. 17.

If the lines of an interaction plot are parallel to each other, then there is no interaction between the two factors, while different sloped lines suggest there might be an interaction present. The interpretation of the results must be taken into consideration with the data analysis from ANOVA results, namely the *p* value. Fig. 17 below shows the interaction plot for each factor. There is a significant difference in the baseline mean value of YM for main effect D:PS (mm/s) regardless of the interaction with all other factors. This plot shows that at 30 mm/s print speed, the mean value is always higher than 20 mm/s print speed regardless of the value of factor A, B or C, Fig. 17.

Focusing on the two interactions where *p* value < 0.05 namely A * B and B * C. Referring to Fig. 17, interaction A * B, there is a significant difference in the baseline mean value of YM for main effect B:layer height regardless of the interaction with A:nozzle temperature. This plot shows that at 0.1 mm layer height the mean value is always higher than 0.2 mm layer height.

The interaction between B:layer height and C:infill density in Fig. 17 shows, the average YM value is reduced with increasing layer height. The result aligns with research stating that voids and improvement in layer bonding occurs at lower layer heights [23]. Interaction B * C shows that at a layer height of 0.1 mm and an infill of 30% or 50% gives a higher mean YM value, of approximately 858 MPa and 870 MPa respectively, than that of the sample with 0.2 mm layer height, 721 MPa for 30% infill and 805 MPa for 50% infill, Fig. 17. This indicates there is a reduction effect at higher layer height level hence YM is dependent on both infill density and layer height. This interaction will be analysed further in T2, section 3.5.

**Fig. 18:**
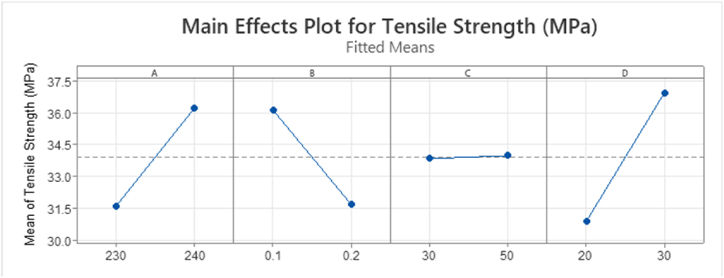
Main Effects Plot for Tensile Strength mean for T1 ANOVA.

**Fig. 19:**
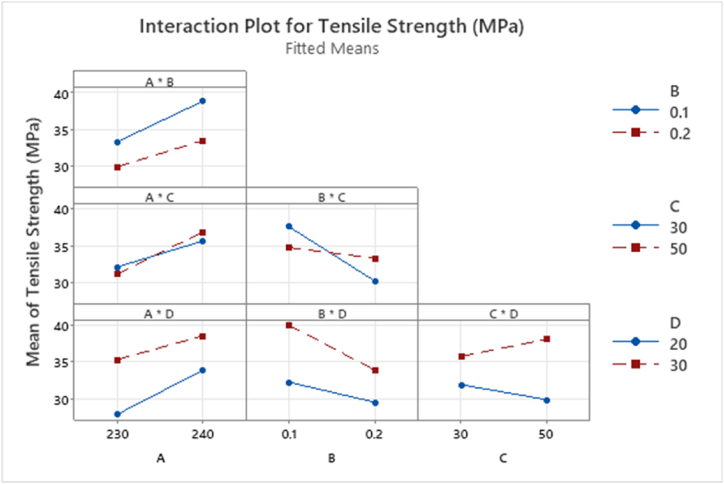
Interaction plot for factorial response data for Tensile Strength (MPa) T1.

Appendix Table 6 highlights in yellow significant main and interaction effects for mean UTS response data. Appendix Table 6 shows that main effects A:NT, B:LH and D:PS and two-way interactions B * C are statistically significant. The analysis shows that factor C:ID is not statistically significant effect on the response mean values. This finding contradicts the results reported in published literature stating an increase in UTS for increasing % infill density [5,17,29,30]. At the lower layer height level, an increase in the response mean value is shown Fig. 18 compared to 0.2 mm layer height, showing alignment with literature results [23,30]. Main effects should not be analysed in isolation hence the interpretation of these results must include the analysis of the interactions plots, Fig. 19.

Fig. 19 below shows the interaction plot for each factor. There is a significant difference in the baseline mean value of UTS for main effect D:PS (mm/s) regardless of the interaction with all other factors. This plot shows that at 30 mm/s print speed, the mean value is always higher than 20 mm/s print speed regardless of the value of factor A, B or C, Fig. 19. The interaction between B:LH and C:ID in Fig. 19 shows that at a layer heightof 0.1 mm and an infill of 30% gives a higher mean UTS value of 37.5 MPa than that of the sample with 50% infill density, 34.5 MPa, Fig. 19. The plot shows a significant decrease in UTS from 0.1 mm layer height (37.7 MPa) to 0.2 mm (30.1 MPa) for 30% infill. The reduction for 50% infill is less than that of 30% infill (34.7 MPa–33.3 MPa). Tensile Strength is dependent on both infill density and layer height. This interaction will be analysed further in T2, section 3.5.

### 3.5 Experimental results for T2

**Fig. 20:**
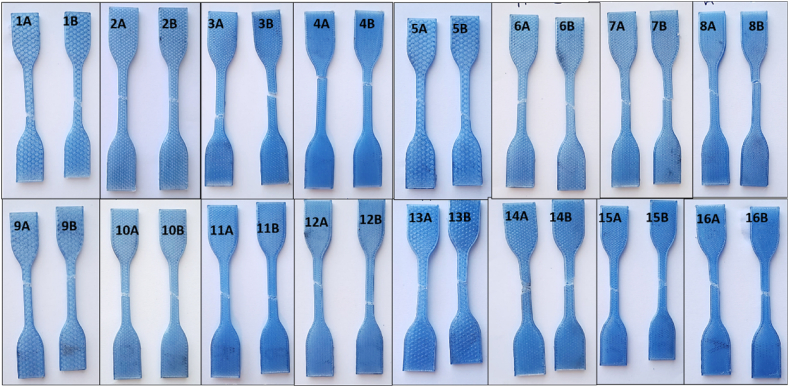
T2 samples after tensile testing.

Appendix Table 7 shows YM and UTS results from T2 tensile testing. Similar to T1 testing, YM values were determined from the average CSA results and UTS from testing each specimen to failure using Avg CSA data. As per Table 3, rPET Ultrafuse® material was used for this set of testing. Fig. 20 shows T2 samples after failure.

**Fig. 21:**
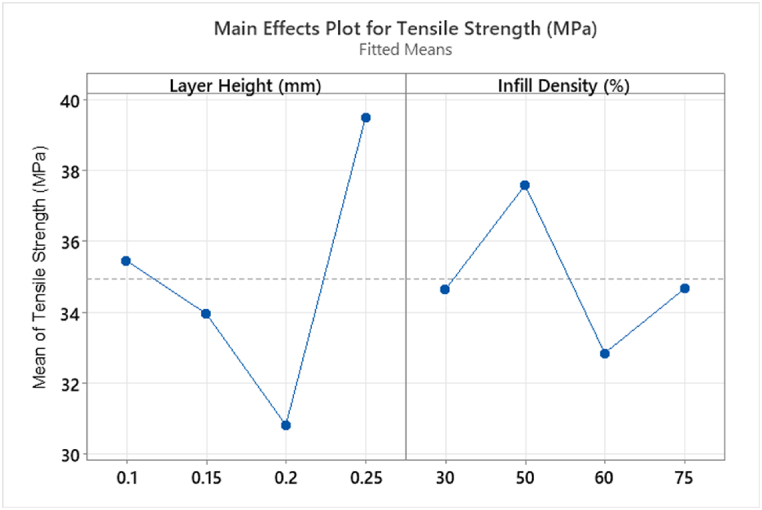
T2 Main Effects Plot for factorial response Tensile Strength (MPa).

ANOVA study was performed (Appendix Table 8) and the resulting main effect plot is shown in Fig. 21. A main effect is present when the mean of the response changes at the different levels of the variable. The steeper the line, the greater the effect it has. Both B:LH and C:ID are shown to be main effects with *p* values < 0.05, Appendix Table 9. Main effect fitted means do not follow reported data from researchers stating an increase in mechanical properties (YM and UTS) with increasing % infill density [5,6,17,27,29,30]. The results presented here indicate a fluctuating trend in UTS with increasing % infill. This finding is influenced by the individual calculated CSA of each sample, taking the CSA profile into account.

**Fig. 22:**
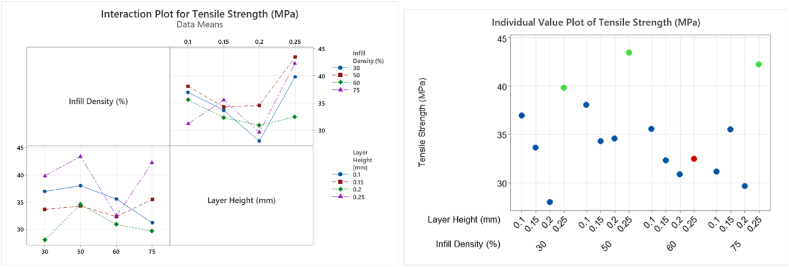
(A) Interaction and (b) Individual plot for response data Tensile Strength (MPa) T2 showing the increase in Tensile Strength at 0.25 mm layer height for all levels except 60% infill density. (Colour is required). (For interpretation of the references to colour in this figure legend, the reader is referred to the Web version of this article.)

The interaction of B:LH and C:ID, B * C, is shown to have a *p* value < 0.05 and determined to be significant in contrary to research by Ref. [24]. As stated previously, main effects should not be analysed in isolation hence the interpretation of these results must include the analysis of the interactions plots, Fig. 22. An interesting observation was noted, rPET_Ultrafuse® material's UTS mean value decreased significantly for 30% infill density at 0.2 mm layer height, from 36.86 MPa to 28.04 MPa (24 % decrease). This decrease was also observed in T1 at 30% infill density under the same interaction B * C but using a different material batch, rPET_1, 37.5 MPa–30.1 MPa (19.7 % decrease) refer to Fig. 17. The most significant increase from B * C interaction can be seen from 0.2 to 0.25 mm layer height, Fig. 22 (a). This is an interesting observation as many published papers detailing layer height for printed parts have seen an opposite effect for different types of filament materials, such as, larger layer height results in low UTS [23,26]. However, the analysis from 0.1 mm to 0.15 mm–0.2 mm aligns with [23] showing a decrease in UTS. Surface roughness and surface finish are also a highlighted concern for larger layer height printed parts [29,34]. Previous research has shown an increase in both YM and UTS when increasing layer height from 0.2 mm to 0.3 mm for PLA printed specimens [29]. However the same increase was not observed from 0.3 mm to 0.4 mm layer height [29]. The results presented here don't follow the same trend. Data shows for all layer heights an increase is seen in UTS from 30% to 50% infill density, Fig. 22. However, a reduction in UTS is shown from 50% to 60% infill, with 0.1 mm and 0.2 mm showing further reduction at 75% infill. Both 0.15 mm and 0.25 mm increase UTS from 60% to 75% infill, Fig. 22. The results presented by Ref. [18] showing a 14% increase in UTS for 20%–80% infill densities is not seen here. Highest values for UTS can be seen at 0.25 mm layer height for all but 60% infill density sample, Fig. 22 (b). These values are in-line with literature PET/rPET UTS ranges 27 MPa–45 MPa [4] and exceed 24 MPa [20].

**Fig. 23:**
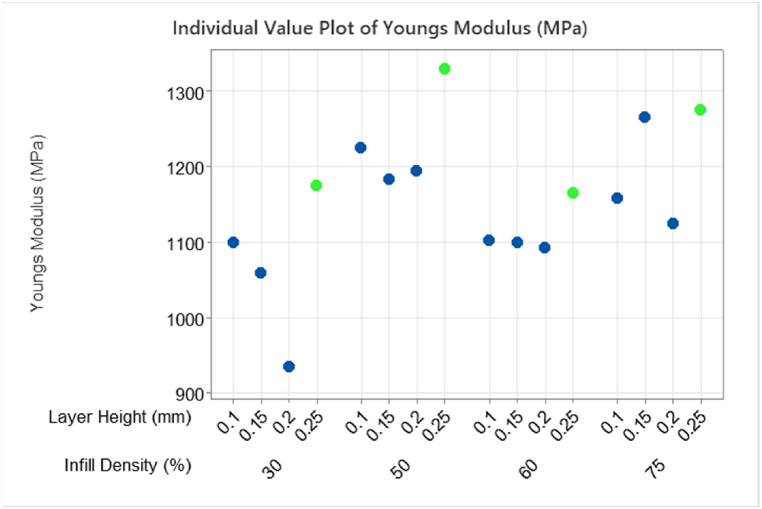
Individual plot of Young's Modulus (MPa) with maximum B:Layer Height (mm) of 0.25 indicated in green for each C:Infill Density (%) level. (Colour is required). (For interpretation of the references to colour in this figure legend, the reader is referred to the Web version of this article.)

Fig. 23 below shows at the larger layer height of 0.25 mm, the average mean values for YM response data for every % infill level, have higher values of YM relative to other layer heights. Printing the part with a 30% infill at a 0.25 mm layer height will produce a part with a similar UTS to printing a part at 60% infill and 0.25 mm layer height. Reducing the infill density can reduce the overall print time. A similar reduction in YM for 0.2 mm and 30% infill density was also seen in T1 analysis.

## 4 Conclusions

This research examined the optimization of 3D printer settings for recycled PET filament material, rPET. The study considered the effect of printer settings; nozzle temperature (°C), layer height (mm), infill density (%), print speed (mm/s) on material properties such as Young's Modulus and Ultimate Tensile Strength. Profile paths for Hexagonal pattern were analysed for various infill densities and layer heights and accurate cross-sectional area (CSA) data were obtained. Cross sectional area analysis showed mean response data was statistically significant if anisotropy was not considered when calculating stress data. On average, a 30% increase was shown for UTS data. This highlights the impact of accurately defining the sample CSA. One significant finding from this study suggests that the thickness of each layer has the most significant impact on the material properties of 3D printed rPET, as observed through the analysis of tensile test data obtained from 3D printed samples. A 3D printed rPET specimen with 30% infill density and 0.25 mm layer height has a higher YM (1175 MPa) and UTS (39 MPa) compared to a specimen with 75% infill density and 0.1 mm layer height (1159 MPa, 31 MPa). However careful interpretation of the results is required because for the same 30% infill parameter at 0.2 mm layer height the YM (936 MPa) and UTM (28 MPa) are significantly lower. If a higher value of YM and UTS is required an infill setting of 50% and layer height of 0.25 mm gave the highest values, YM:1330 MPa and UTS 43 MPa. The UTS results in this research under the optimal settings is higher than the material data sheet provided from the material manufacture, stating UTS 38.6 MPa. The advantage of using recycled filament materials are numerous and the data from this study shows material properties of rPET such as UTS 43 MPa are in line and above UTS for virgin filament materials such as PET: 33.4 MPa; ABS: 36 MPa; PLA: 34.7 MPa. Whereas YM of analysed rPET sample at optimal settings (240 °C, 0.25 mm, 50%, 30 mm/s) is shown to be 1330 MPa which is below reported values for virgin filament materials such as PET: 1662 MPa; ABS: 1833 MPa; PLA: 1860 MPa. rPET filament is an excellent alternative and sustainable material for 3D printing and optimizing printer settings can align the user requirements and printed part properties. Further studies on the degradation of material properties of rPET filament after multiple recycling cycles should be examined along with studies on practical applications.

## Data availability statement

The data associated with our work has not been deposited into a publicly available repository. The data will be available on request.

## CRediT authorship contribution statement

**Ciara O'Driscoll:** Writing – review & editing, Writing – original draft, Supervision, Methodology, Funding acquisition, Formal analysis, Data curation, Conceptualization. **Olamide Owodunni:** Investigation. **Umar Asghar:** Writing – review & editing, Conceptualization.

## Declaration of competing interest

The authors declare that they have no known competing financial interests or personal relationships that could have appeared to influence the work reported in this paper.

## Figures and Tables

**Table 1:** CSA profile data for T1 samples A and B. All data values are in mm^2^.

Number	Sample A CSA Profiles				Sample B CSA Profiles			
	Measured	Max	Min	Avg	Measured	Max	Min	Avg
**1**	20.3	15.98	13.58	14.78	20	15.68	13.28	14.48
**2**	20.2	15.88	13.48	14.68	20.48	16.16	13.76	14.96
**3**	20.1	14.1	13.14	13.62	19.55	13.55	12.59	13.07
**4**	20.4	14.4	13.44	13.92	20.48	14.48	13.52	14
**5**	19.5	16.62	15.02	15.82	20.1	17.22	15.62	16.42
**6**	20.3	17.42	15.82	16.62	20.3	17.42	15.82	16.62
**7**	20.2	16.2	15.56	15.88	19.65	15.65	15.01	15.33
**8**	20.4	16.4	15.76	16.08	20.3	16.3	15.66	15.98
**9**	20.4	16.08	13.68	14.88	20.3	15.98	13.58	14.78
**10**	20.5	16.18	13.78	14.98	20.18	15.86	13.46	14.66
**11**	19.4	13.4	12.44	12.92	19.56	13.56	12.6	13.08
**12**	20.6	14.6	13.64	14.12	20.3	14.3	13.34	13.82
**13**	20.1	17.22	15.62	16.42	19.65	16.77	15.17	15.97
**14**	20.1	17.22	15.62	16.42	20.31	17.43	15.83	16.63
**15**	19.9	15.9	15.26	15.58	19.77	15.77	15.13	15.45
**16**	20.3	16.3	15.66	15.98	20.29	16.29	15.65	15.97

**Table 2:** CSA profile data for T2 samples A and B. All data values are in mm^2^.

Sample	Sample A CSA Profiles				Sample B CSA Profiles			
number	Measured	Max	Min	Avg	Measured	Max	Min	Avg
1	20.11	15.79	12.93	14.36	20.24	16.83	13.06	14.49
2	20.24	14.24	13.28	13.76	19.60	14.64	12.64	13.12
3	20.00	16.64	14.96	15.80	19.60	17.04	14.56	15.40
4	20.30	17.42	15.50	16.46	20.17	18.39	15.37	16.33
5	20.15	16.55	14.55	15.55	20.05	17.40	14.45	15.45
6	20.34	15.34	14.54	14.94	20.40	16.54	14.60	15.00
7	20.12	17.32	15.92	16.62	19.86	17.98	15.66	16.36
8	20.01	17.61	14.01	15.81	20.05	18.46	14.05	15.85
9	20.07	17.19	15.59	16.39	20.01	18.00	15.53	16.33
10	19.75	15.75	15.11	15.43	19.80	16.35	15.16	15.48
11	19.97	17.73	16.61	17.17	19.85	18.38	16.49	17.05
12	20.08	18.16	16.88	17.52	20.10	19.06	16.90	17.54
13	20.52	18.36	17.16	17.76	20.50	19.66	17.14	17.74
14	20.68	17.68	17.20	17.44	20.33	18.81	16.85	17.09
15	20.65	18.97	18.13	18.55	20.51	20.28	17.99	18.41
16	20.18	18.74	17.78	18.26	20.22	19.76	17.82	18.30

**Table 3:** The calculated Young's Modulus data (MPa) for specimens under four main testing conditions: firstly, measured external CSA dimensions; secondly, maximum calculated CSA; thirdly, minimum calculated CSA; finally, average calculated CSA.

Sample	Measured CSA			Max CSA			Min CSA			Avg CSA		
Number	1	2	3	1	2	3	1	2	3	1	2	3
1	662	669	670	749	776	781	881	914	919	809	839	844
2	730	740	740	840	866	870	990	1020	1025	909	937	941
3	653	658	658	827	853	854	887	916	917	856	883	884
4	746	754	754	926	980	985	992	1051	1056	958	1014	1020
5	610	617	618	633	655	657	700	724	727	665	688	691
6	684	692	690	720	743	746	792	818	821	754	778	781
7	592	600	601	655	677	681	682	704	709	669	690	695
8	738	748	748	837	860	865	871	895	900	853	878	882
9	601	606	607	671	696	698	789	818	821	725	752	755
10	739	742	746	848	873	877	995	1025	1030	916	942	947
11	496	–	–	678	–	–	730	–	–	702	–	–
12	703	709	709	888	913	916	950	978	981	918	944	948
13	610	611	610	631	651	653	696	718	720	661	682	684
14	672	679	679	698	722	726	769	796	800	732	758	761
15	671	674	673	745	768	776	776	801	808	760	784	792
16	731	734	734	806	833	837	841	869	873	826	853	857
												

**Table 4:** ANOVA results showing F and P values for Sample 1A

Method	
Null hypothesis	All means are equal
Alternative hypothesis	Not all means are equal
Significance level	α = 0.05

Factor Information		
Factor	Levels	Values
CSA	4	AVG, MAX, Measured, MIN

Sample 1: Analysis of Variance
Source DF Adj SS Adj MS F-Value *P*-Value
CSA 3 178903 59634.3204.21 0.000
Error 20 5840 292.0
Total 23 184 743

Sample 1: Grouping Information Using the Tukey Method and 95% Confidence						
CSA	N	Mean	Grouping			
MIN	6	896.7	A			
AVG	6	823.14		B		
MAX	6	760.74			C	
Measured	6	661.31				D

Means that do not share a letter are significantly different.

Equal variances were assumed for the analysis.

**Table 5:** ANOVA results for T1 Young's Modulus mean analysis

Model Summary			
S	*R*-sq	*R*-sq (adj)	*R*-sq (pred)
34.4072	93.13%	89.35%	82.41%

Analysis of Variance					
Source	DF	Adj SS	Adj MS	F-Value	*P*-Value
Model	11	320 924	29 175	24.64	0
Blocks	1	39	39	0.03	0.857
Linear	4	286 943	71 736	60.59	0
A	1	18 038	18 038	15.24	0.001
B	1	80 434	80 434	67.94	0.000
C	1	18 120	18 120	15.31	0.001
D	1	170 351	170 351	143.9	0.000
2-Way Interactions	6	33 942	5657	4.78	0.004
A * B	1	12 400	12 400	10.47	0.004
A * C	1	1121	1121	0.95	0.342
A * D	1	943	943	0.8	0.383
B * C	1	10 620	10 620	8.97	0.007
B * D	1	4648	4648	3.93	0.061
C * D	1	4210	4210	3.56	0.074
Error	20	23 677	1184		
Total	31	344 602			

**Table 6:** ANOVA results for T1 using Tensile Strength values as the response data. Yellow highlighted main effects and interactions are significantly relevant with *p* values < 0.05.

Model Summary			
S	*R*-sq	*R*-sq (adj)	*R*-sq (pred)
3.31677	78.15%	66.14%	44.08%

Analysis of Variance					
Source	DF	Adj SS	Adj MS	F-Value	*P*-Value
Model	11	787.15	71.56	6.5	0.000
Blocks	1	8.02	8.02	0.73	0.403
Linear	4	615.13	153.783	13.98	0.000
A	1	169.46	169.464	15.4	0.001
B	1	155.94	155.938	14.17	0.001
C	1	0.17	0.168	0.02	0.903
D	1	289.56	289.562	26.32	0.000
2-Way Interactions	6	164	27.334	2.48	0.058
A*B	1	7.45	7.45	0.68	0.420
A * C	1	9.37	9.374	0.85	0.367
A * D	1	15.54	15.54	1.41	0.249
B * C	1	70.92	70.924	6.45	0.020
B * D	1	23.09	23.086	2.1	0.163
C * D	1	37.63	37.628	3.42	0.079
Error	20	220.02	11.001		
Total	31	1007.17			

**Table 7:** T2 Young's Modulus (MPa) and Tensile Strength response results

Sample number			Response variables:			
			Young's Modulus (MPa)		Tensile Strength (MPa)	
	B (mm)	C (%)	Specimen a mean value	Specimen b mean value	Specimen a	Specimen b
1	0.1	30	1128.63	1073.10	35.55	38.41
2	0.1	50	1162.10	1289.93	35.00	41.07
3	0.1	60	1120.17	1086.87	34.70	36.50
4	0.1	75	1152.47	1165.87	30.20	32.20
5	0.15	30	1020.45	1100.47	31.47	35.86
6	0.15	50	1228.93	1137.83	34.46	34.14
7	0.15	60	1034.10	1167.60	30.20	34.50
8	0.15	75	1262.10	1270.57	34.68	36.36
9	0.2	30	904.99	967.32	26.08	30.01
10	0.2	50	1219.70	1170.30	35.31	33.85
11	0.2	60	1059.30	1126.87	28.46	33.35
12	0.2	75	1152.87	1096.87	28.20	31.12
13	0.25	30	1161.90	1188.73	39.92	39.76
14	0.25	50	1332.03	1329.57	43.40	43.47
15	0.25	60	1146.10	1184.17	30.67	34.29
16	0.25	75	1283.20	1269.40	43.68	40.86

**Table 8:** ANOVA results table for T2 Tensile Strength (MPa) data

Factor Information		
Factor	Levels	Values
Layer Height (mm)	4	0.1, 0.15, 0.2, 0.25
Infill Density (%)	4	30, 50, 60, 75

Analysis of Variance					
Source	DF	Adj SS	Adj MS	F-Value	*P*-Value
Model	16	605.69	37.856	12.24	0.000
Blocks	1	35.64	35.638	11.52	0.004
Linear	6	406.83	67.805	21.93	0.000
Layer Height (mm)	3	313.90	104.633	33.83	0.000
Infill Density (%)	3	92.93	30.978	10.02	0.001
2-Way Interactions	9	163.22	18.136	5.86	0.001
Layer Height (mm)*Infill Density (%)	9	163.22	18.136	5.86	0.001
Error	15	46.39	3.093		
Total	31	652.08			

Model Summary			
S	*R*-sq	*R*-sq (adj)	*R*-sq (pred)
1.75858	92.89%	85.30%	67.62%

## Footnotes

1 rPET_1 material manufacturing process is not included in this paper however preliminary testing was conducted on the filament material to determine and verify material property values fell within acceptable rPET ranges as per literature.

2 rPET Ultrafuse® specimen was printed flat (x-y plane), material property data sheet is available [21]. B.D.P.S. BV, Technical Data sheet Ultrafuse® rPET, 2023. https://move.forward-am.com/hubfs/AES%20Documentation/Standard%20Filaments/rPET/TDS/Ultrafuse_rPET_TDS_EN_v3.2.pdf.
